# Supplementary material for: Mechanical breathing in organic electrochromics
Source: Nat Commun. 2020 Jan 10;11:211. doi: 10.1038/s41467-019-14047-8 (PMC6954196; doi:10.1038/s41467-019-14047-8)
Supplement: Supplementary file 1 — Supplementary Information [file 41467_2019_14047_MOESM1_ESM.pdf]

Mechanical Breathing in Organic Electrochromics

Wang et al.

Supplementary Information for

Mechanical Breathing in Organic Electrochromics

Xiaokang Wang, Ke Chen, Luize Scalco de Vasconcelos, Jiazhi He, Yung C. Shin, Jianguo Mei, and Kejie Zhao

This document contains supplementary information for *Mechanical Breathing in Organic Electrochromics*, including Supplementary Figures, Supplementary Table, and Supplementary Note. Supplementary Movie 1 entitled "Mechanical breathing of the PProDOT thin film on ITO electrode" can be found at http://doi.org/10.17605/OSF.IO/5Y9AM .

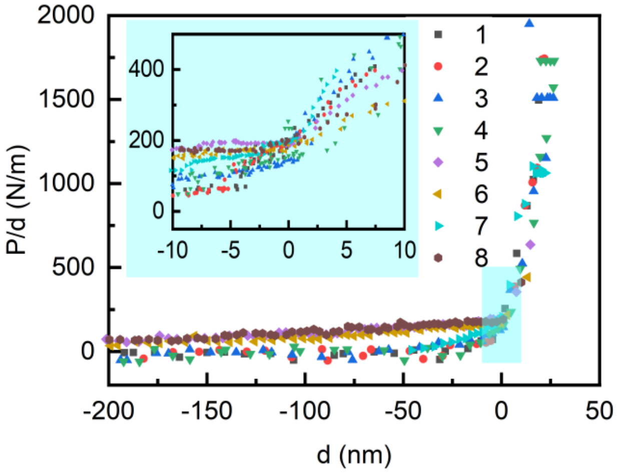

Supplementary Figure 1. The slope (P/d) of load-displacement in the nanoindentation test when the tip is approaching the surface of the thin film. The abrupt change in slope indicates the surface detection.

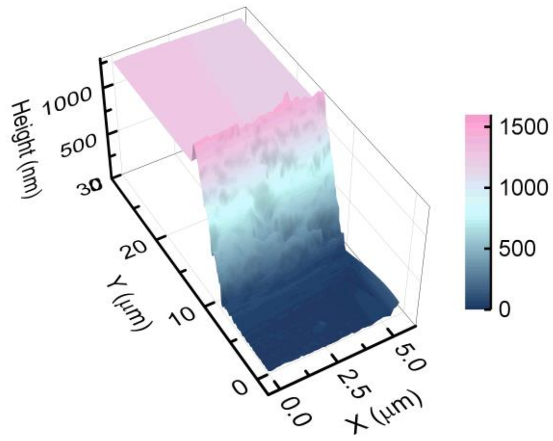

Supplementary Figure 2. AFM image of the PProDOT thin film. Average thickness is 1222.0 ± 1.0 nm. Customized indentation at the same location gives an average thickness of 1278.5 ± 92.9 nm.

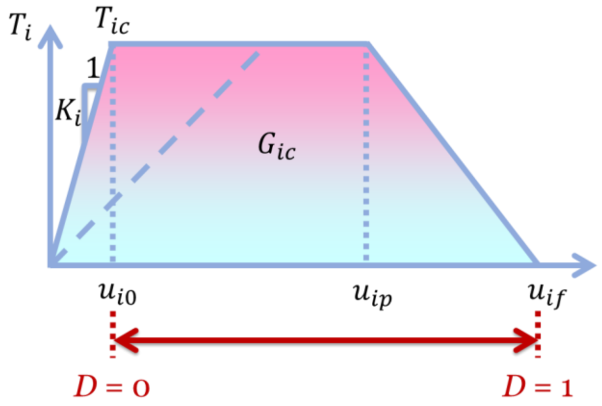

Supplementary Figure 3. The traction-separation constitutive law to describe the damage initiation and crack growth at the interface. The traction force linearly increases upon reaching the maximum value T_{ic} at a displacement of u_{i0}.The traction maintains a constant value to mimic the plastic behavior at the interface, and then decreases linearly to zero at u_{if} when the energy dissipated is equal to the interfacial toughness G_{ic}. i = I (II) in case of mode-I (II) crack. The interface damage initiates at u_{i0} (D = 0) while crack opens at u_{if} (D = 1). Unloading follows the dash line with reduced stiffness.

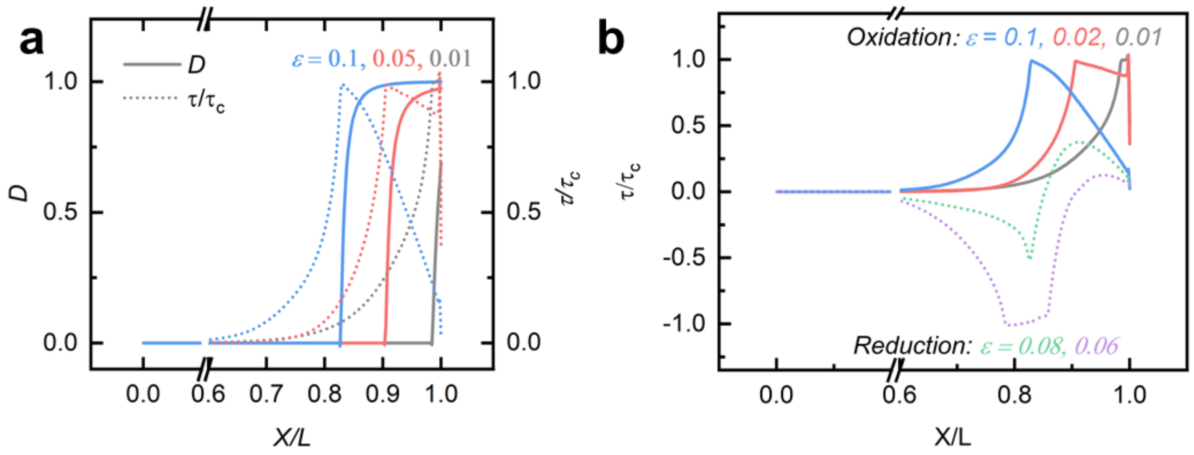

Supplementary Figure 4. Evolution of stress and damage along the interface during 1st cycle. **a** The damage function (solid lines) and shear stress profile (dotted lines) at the interface when the thin film is subject to various strains in the first oxidation reaction. **b** The shear stress profile at the interface when the thin film is subject to various strains in the first oxidation reaction (solid lines) and first reduction reaction (dotted lines).

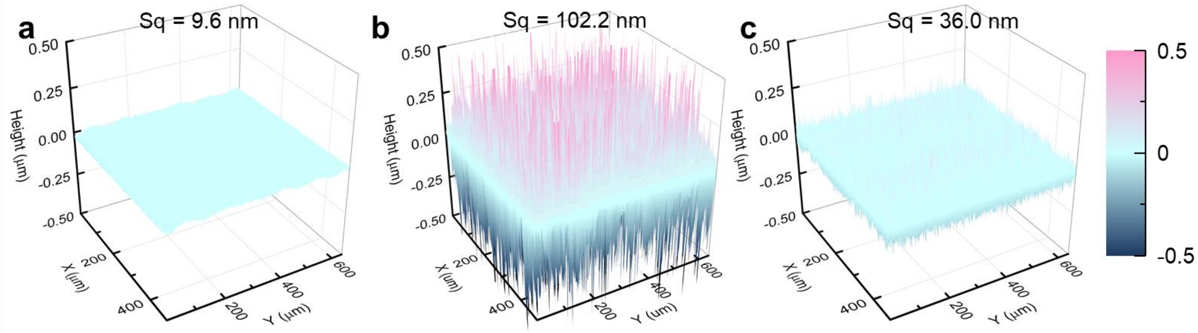

Supplementary Figure 5. Surface profile of the ITO surface via optical surface profilometer. **a** Bare ITO. **b** Roughened ITO. **c** SiNP treated ITO. Sq denotes the root mean square height of the surface.

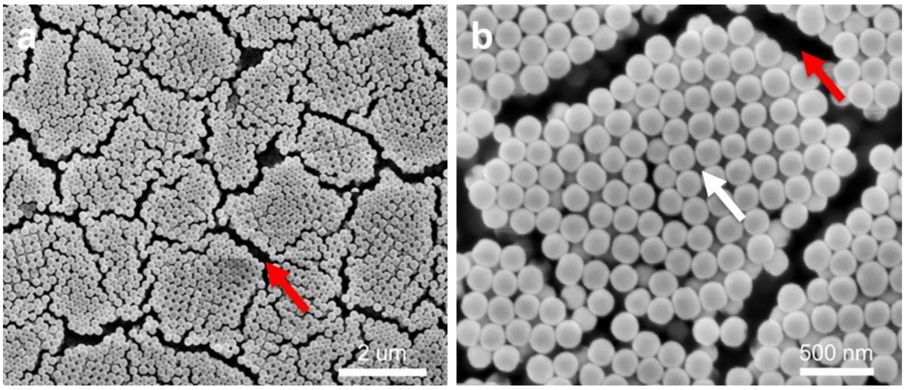

Supplementary Figure 6. Scanning electron microscopy images of SiO_2 nanoparticles deposited on ITO-glass substrate. Scale bar is **a** 2um and **b** 500nm, respectively. White arrow indicates interparticle gaps. Red arrows indicate mud cracks induced by electron-wind forces during SEM imaging.

Supplementary Table 1. The thin film thickness measurements by AFM and nanoindentation.

Site No.	AFM (nm)	Nanoindenter (nm)
1	925.3 ± 3.8	991.8 ± 67.6
2	1222.0 ± 1.0	1278.5 ± 92.9
3	1198.8 ± 2.3	1140.8 ± 183.4
4	1121.1 ± 1.6	1005.4 ± 94.6

Supplementary Note 1

Developed by Dugdale (1960) and Barenblatt (1962)[1,2], cohesive zone model is widely used in modelling crack initiation and propagation. A trapezoidal traction-separation law, as described in Fig. S3, shows that when the relative displacement u_i of the points in contact increases, the traction between the contacting surfaces increases linearly with a stiffness K_i before reaching the maximum value T_{ic} at u_{i0}, followed by a constant traction. i can be I (II) to denote normal(tangential) direction, and $K_i = \frac{E_{\text{film}}}{\min(\textit{mesh size})}$ where E_{film} is the modulus of the film. We set maximum normal traction the same value as yield stress σ_{Y}, and maximum shear traction as $\sigma_{\text{Y}}/\sqrt{6}$ such that damage initiates once plastic flow occur in the mode-II fracture. Young's(shear) modulus is used as E_{film} for normal (shear) stiffness in our simulation. The interface is damaged ($D>0$) when maximum traction is reached and fails ($D=1$) when the energy dissipated equals the interfacial toughness G_{ic} (shaded area in Fig. S3) at a relative displacement of u_{if}. For trapezoidal traction-separation law, a factor λ, defined as the ratio between the plastic (constant traction) part and the total inelastic part, is used to characterize the shape of the trapezoid. $\lambda = 0.75$ is used in the modelling[3,4].

For mixed mode cracking, we define the displacement

$$u = \sqrt{\langle u_{\text{I}} \rangle^2 + u_{\text{II}}^2} \tag{1}$$

where

$$\langle x \rangle = \begin{cases} x & x \geq 0 \\ 0 & x < 0 \end{cases}$$

Damage initiates when the relative displacement satisfy

$$u_0 = u_{\text{I0}} u_{\text{II0}} \sqrt{\frac{u^2}{\langle u_{\text{I}} \rangle^2 u_{\text{II0}}^2 + u_{\text{II}}^2 u_{\text{I0}}^2}} \tag{2}$$

The mixed mode failure displacement is

$$u_{\text{f}} = 2G_{\text{Ic}} G_{\text{IIc}} \frac{1+\beta^2}{u_0 \left(K_{\text{I}} G_{\text{IIc}} + \beta^2 K_{\text{II}} G_{\text{Ic}} \right)} - \left(u_{\text{p}} - u_0 \right) \tag{3}$$

where

$$u_{\text{p}} = u_{\text{Ip}} u_{\text{IIp}} \sqrt{\frac{u^2}{\langle u_{\text{I}} \rangle^2 u_{\text{IIp}}^2 + u_{\text{II}}^2 u_{\text{Ip}}^2}} \tag{4}$$

and

$$\beta = \frac{u_{\text{II}}}{u_{\text{I}}}. \tag{5}$$

A linear mixed mode failure law is assumed where

$$\frac{G_{\text{I}}}{G_{\text{Ic}}} + \frac{G_{\text{II}}}{G_{\text{IIc}}} = 1 \tag{6}$$

The damage function can be written as

$$D(u) = \min\left(1, \max\left(0, \begin{cases} 1 - \dfrac{u_0}{u} & u < u_\mathrm{p} \\ 1 - \dfrac{u_0}{u}\left(\dfrac{u_\mathrm{f} - u}{u_\mathrm{f} - u_\mathrm{p}}\right) & u \geq u_\mathrm{p} \end{cases}\right)\right) \quad (7)$$

Note that we modified the embedded code for calculating damage function via a variable monitoring the maximum extent of damage. This allows us to perform cyclic (non-monotonous) loading.

Supplementary References

1. Dugdale, D. S. Yielding of steel sheets containing slits. *Journal of the Mechanics and Physics of Solids* **8**, 100–104 (1960).
2. Barenblatt, G. I. The Mathematical Theory of Equilibrium Cracks in Brittle Fracture. in *Advances in Applied Mechanics* (eds. Dryden, H. L., von Kármán, Th., Kuerti, G., van den Dungen, F. H. & Howarth, L.) **7**, 55–129 (Elsevier, 1962).
3. Tvergaard, V. & Hutchinson, J. W. The relation between crack growth resistance and fracture process parameters in elastic-plastic solids. *Journal of the Mechanics and Physics of Solids* **40**, 1377–1397 (1992).
4. Chandra, N., Li, H., Shet, C. & Ghonem, H. Some issues in the application of cohesive zone models for metal–ceramic interfaces. *International Journal of Solids and Structures* **39**, 2827–2855 (2002).
