# Supplementary material for: Mechanical breathing in organic electrochromics
Source: Nat Commun. 2020 Jan 10;11:211. doi: 10.1038/s41467-019-14047-8 (PMC6954196; doi:10.1038/s41467-019-14047-8)
Supplement: Supplementary file 3 — Description of Additional Supplementary Files [file 41467_2019_14047_MOESM3_ESM.pdf]

Description of Additional Supplementary Files

File Name: Supplementary Movie 1
Description: Mechanical breathing of the PProDOT thin film on ITO electrode
